# Intraarticular steroids as DMARD-sparing agents for juvenile idiopathic arthritis flares: Analysis of the Childhood Arthritis and Rheumatology Research Alliance Registry

**DOI:** 10.1186/s12969-022-00770-y

**Published:** 2022-11-25

**Authors:** Timothy Hahn, Carrie Daymont, Timothy Beukelman, Brandt Groh, Kimberly Hays, Catherine April Bingham, Lisabeth Scalzi, for the CARRA Registry investigators, N. Abel, K. Abulaban, A. Adams, M. Adams, R. Agbayani, J. Aiello, S. Akoghlanian, C. Alejandro, E. Allenspach, R. Alperin, M. Alpizar, G. Amarilyo, W. Ambler, E. Anderson, S. Ardoin, S. Armendariz, E. Baker, I. Balboni, S. Balevic, L. Ballenger, S. Ballinger, N. Balmuri, F. Barbar-Smiley, L. Barillas-Arias, M. Basiaga, K. Baszis, M. Becker, H. Bell-Brunson, E. Beltz, H. Benham, S. Benseler, W. Bernal, T. Beukelman, T. Bigley, B. Binstadt, C. Black, M. Blakley, J. Bohnsack, J. Boland, A. Boneparth, S. Bowman, C. Bracaglia, E. Brooks, M. Brothers, A. Brown, H. Brunner, M. Buckley, M. Buckley, H. Bukulmez, D. Bullock, B. Cameron, S. Canna, L. Cannon, P. Carper, V. Cartwright, E. Cassidy, L. Cerracchio, E. Chalom, J. Chang, A. Chang-Hoftman, V. Chauhan, P. Chira, T. Chinn, K. Chundru, H. Clairman, D. Co, A. Confair, H. Conlon, R. Connor, A. Cooper, J. Cooper, S. Cooper, C. Correll, R. Corvalan, D. Costanzo, R. Cron, L. Curiel-Duran, T. Curington, M. Curry, A. Dalrymple, A. Davis, C. Davis, C. Davis, T. Davis, F. De Benedetti, D. De Ranieri, J. Dean, F. Dedeoglu, M. DeGuzman, N. Delnay, V. Dempsey, E. DeSantis, T. Dickson, J. Dingle, B. Donaldson, E. Dorsey, S. Dover, J. Dowling, J. Drew, K. Driest, Q. Du, K. Duarte, D. Durkee, E. Duverger, J. Dvergsten, A. Eberhard, M. Eckert, K. Ede, B. Edelheit, C. Edens, C. Edens, Y. Edgerly, M. Elder, B. Ervin, S. Fadrhonc, C. Failing, D. Fair, M. Falcon, L. Favier, S. Federici, B. Feldman, J. Fennell, I. Ferguson, P. Ferguson, B. Ferreira, R. Ferrucho, K. Fields, T. Finkel, M. Fitzgerald, C. Fleming, O. Flynn, L. Fogel, E. Fox, M. Fox, L. Franco, M. Freeman, K. Fritz, S. Froese, R. Fuhlbrigge, J. Fuller, N. George, K. Gerhold, D. Gerstbacher, M. Gilbert, M. Gillispie-Taylor, E. Giverc, C. Godiwala, I. Goh, H. Goheer, D. Goldsmith, E. Gotschlich, A. Gotte, B. Gottlieb, C. Gracia, T. Graham, S. Grevich, T. Griffin, J. Griswold, A. Grom, M. Guevara, P. Guittar, M. Guzman, M. Hager, T. Hahn, O. Halyabar, E. Hammelev, M. Hance, A. Hanson, L. Harel, S. Haro, J. Harris, O. Harry, E. Hartigan, J. Hausmann, A. Hay, K. Hayward, J. Heiart, K. Hekl, L. Henderson, M. Henrickson, A. Hersh, K. Hickey, P. Hill, S. Hillyer, L. Hiraki, M. Hiskey, P. Hobday, C. Hoffart, M. Holland, M. Hollander, S. Hong, M. Horwitz, J. Hsu, A. Huber, J. Huggins, J. Hui-Yuen, C. Hung, J. Huntington, A. Huttenlocher, M. Ibarra, L. Imundo, C. Inman, A. Insalaco, A. Jackson, S. Jackson, K. James, G. Janow, J. Jaquith, S. Jared, N. Johnson, J. Jones, J. Jones, J. Jones, K. Jones, S. Jones, S. Joshi, L. Jung, C. Justice, A. Justiniano, N. Karan, K. Kaufman, A. Kemp, E. Kessler, U. Khalsa, B. Kienzle, S. Kim, Y. Kimura, D. Kingsbury, M. Kitcharoensakkul, T. Klausmeier, K. Klein, M. Klein-Gitelman, B. Kompelien, A. Kosikowski, L. Kovalick, J. Kracker, S. Kramer, C. Kremer, J. Lai, J. Lam, B. Lang, S. Lapidus, B. Lapin, A. Lasky, D. Latham, E. Lawson, R. Laxer, P. Lee, P. Lee, T. Lee, L. Lentini, M. Lerman, D. Levy, S. Li, S. Lieberman, L. Lim, C. Lin, N. Ling, M. Lingis, M. Lo, D. Lovell, D. Lowman, N. Luca, S. Lvovich, C. Madison, J. Madison, S. Magni Manzoni, B. Malla, J. Maller, M. Malloy, M. Mannion, C. Manos, L. Marques, A. Martyniuk, T. Mason, S. Mathus, L. McAllister, K. McCarthy, K. McConnell, E. McCormick, D. McCurdy, P. Mc Curdy Stokes, S. McGuire, I. McHale, A. McMonagle, C. McMullen-Jackson, E. Meidan, E. Mellins, E. Mendoza, R. Mercado, A. Merritt, L. Michalowski, P. Miettunen, M. Miller, D. Milojevic, E. Mirizio, E. Misajon, M. Mitchell, R. Modica, S. Mohan, K. Moore, L. Moorthy, S. Morgan, E. Morgan Dewitt, C. Moss, T. Moussa, V. Mruk, A. Murphy, E. Muscal, R. Nadler, B. Nahal, K. Nanda, N. Nasah, L. Nassi, S. Nativ, M. Natter, J. Neely, B. Nelson, L. Newhall, L. Ng, J. Nicholas, R. Nicolai, P. Nigrovic, J. Nocton, B. Nolan, E. Oberle, B. Obispo, B. O’Brien, T. O’Brien, O. Okeke, M. Oliver, J. Olson, K. O’Neil, K. Onel, A. Orandi, M. Orlando, S. Osei-Onomah, R. Oz, E. Pagano, A. Paller, N. Pan, S. Panupattanapong, M. Pardeo, J. Paredes, A. Parsons, J. Patel, K. Pentakota, P. Pepmueller, T. Pfeiffer, K. Phillippi, D. Pires Marafon, K. Phillippi, L. Ponder, R. Pooni, S. Prahalad, S. Pratt, S. Protopapas, B. Puplava, J. Quach, M. Quinlan-Waters, C. Rabinovich, S. Radhakrishna, J. Rafko, J. Raisian, A. Rakestraw, C. Ramirez, E. Ramsay, S. Ramsey, R. Randell, A. Reed, A. Reed, A. Reed, H. Reid, K. Remmel, A. Repp, A. Reyes, A. Richmond, M. Riebschleger, S. Ringold, M. Riordan, M. Riskalla, M. Ritter, R. Rivas-Chacon, A. Robinson, E. Rodela, M. Rodriquez, K. Rojas, T. Ronis, M. Rosenkranz, B. Rosolowski, H. Rothermel, D. Rothman, E. Roth-Wojcicki, K. Rouster-Stevens, T. Rubinstein, N. Ruth, N. Saad, S. Sabbagh, E. Sacco, R. Sadun, C. Sandborg, A. Sanni, L. Santiago, A. Sarkissian, S. Savani, L. Scalzi, L. Schanberg, S. Scharnhorst, K. Schikler, A. Schlefman, H. Schmeling, K. Schmidt, E. Schmitt, R. Schneider, K. Schollaert-Fitch, G. Schulert, T. Seay, C. Seper, J. Shalen, R. Sheets, A. Shelly, S. Shenoi, K. Shergill, J. Shirley, M. Shishov, C. Shivers, E. Silverman, N. Singer, V. Sivaraman, J. Sletten, A. Smith, C. Smith, J. Smith, J. Smith, E. Smitherman, J. Soep, M. Son, S. Spence, L. Spiegel, J. Spitznagle, R. Sran, H. Srinivasalu, H. Stapp, K. Steigerwald, Y. Sterba Rakovchik, S. Stern, A. Stevens, B. Stevens, R. Stevenson, K. Stewart, C. Stingl, J. Stokes, M. Stoll, E. Stringer, S. Sule, J. Sumner, R. Sundel, M. Sutter, R. Syed, G. Syverson, A. Szymanski, S. Taber, R. Tal, A. Tambralli, A. Taneja, T. Tanner, S. Tapani, G. Tarshish, S. Tarvin, L. Tate, A. Taxter, J. Taylor, M. Terry, M. Tesher, A. Thatayatikom, B. Thomas, K. Tiffany, T. Ting, A. Tipp, D. Toib, K. Torok, C. Toruner, H. Tory, M. Toth, S. Tse, V. Tubwell, M. Twilt, S. Uriguen, T. Valcarcel, H. Van Mater, L. Vannoy, C. Varghese, N. Vasquez, K. Vazzana, R. Vehe, K. Veiga, J. Velez, J. Verbsky, G. Vilar, N. Volpe, E. von Scheven, S. Vora, J. Wagner, L. Wagner-Weiner, D. Wahezi, H. Waite, J. Walker, H. Walters, T. Wampler Muskardin, L. Waqar, M. Waterfield, M. Watson, A. Watts, P. Weiser, J. Weiss, P. Weiss, E. Wershba, A. White, C. Williams, A. Wise, J. Woo, L. Woolnough, T. Wright, E. Wu, A. Yalcindag, M. Yee, E. Yen, R. Yeung, K. Yomogida, Q. Yu, R. Zapata, A. Zartoshti, A. Zeft, R. Zeft, Y. Zhang, Y. Zhao, A. Zhu, C. Zic

**Affiliations:** 1 Department of Pediatrics, Penn State Children’s Hospital, 500 University Dr, Hershey, 90 Hope Drive, P.O. Box 855, Hershey, PA 17033-0855 USA; 2 Department of Pediatrics, University of Alabama at Birmingham, CPPN G10, 1600 7th Ave South, Birmingham, AL 35233 USA

**Keywords:** Juvenile idiopathic arthritis (JIA), Steroids, Intraarticular steroid injections, Pediatric rheumatology

## Abstract

**Background:**

Children with juvenile idiopathic arthritis (JIA) who achieve a drug free remission often experience a flare of their disease requiring either intraarticular steroids (IAS) or systemic treatment with disease modifying anti-rheumatic drugs (DMARDs). IAS offer an opportunity to recapture disease control and avoid exposure to side effects from systemic immunosuppression. We examined a cohort of patients treated with IAS after drug free remission and report the probability of restarting systemic treatment within 12 months.

**Methods:**

We analyzed a cohort of patients from the Childhood Arthritis and Rheumatology Research Alliance (CARRA) Registry who received IAS for a flare after a period of drug free remission. Historical factors and clinical characteristics and of the patients including data obtained at the time of treatment were analyzed.

**Results:**

We identified 46 patients who met the inclusion criteria. Of those with follow up data available 49% had restarted systemic treatment 6 months after IAS injection and 70% had restarted systemic treatment at 12 months. The proportion of patients with prior use of a biologic DMARD was the only factor that differed between patients who restarted systemic treatment those who did not, both at 6 months (79% vs 35%, *p* < 0.01) and 12 months (81% vs 33%, *p* < 0.05).

**Conclusion:**

While IAS are an option for all patients who flare after drug free remission, it may not prevent the need to restart systemic treatment. Prior use of a biologic DMARD may predict lack of success for IAS. Those who previously received methotrexate only, on the other hand, are excellent candidates for IAS.

## Background

In the treatment of non-systemic forms of Juvenile Idiopathic Arthritis (JIA) the initial treatment is based, in part, on the number joints with active arthritis [1]. Children with more than 4 joints involved (polyarticular JIA) generally receive systemic treatment with methotrexate or a biologic disease modifying anti-rheumatic drug (DMARD), whereas children with less than or equal to 4 joints involved (oligoarticular JIA) generally are treated with intraarticular steroid (IAS) injection. If oligoarticular arthritis is refractory to IAS or disease relapses within a short interval, systemic treatment is needed.

This recommendation to use IAS for children with a few joints involved is based on a desire to minimize toxicity and cost associated with systemic treatment while minimizing joint damage and long term morbidity. Methotrexate intolerance, which primarily manifests as nausea and vomiting, occurs in up to 50% of children and is associated with decreased quality of life [2–6]. The prevalence of methotrexate intolerance appears to increase with prolonged duration of treatment and occurs regardless of the route of delivery (oral or subcutaneous). Many patients require treatment with pharmacologic and/or non-pharmacologic interventions but the efficacy of these interventions varies and many patients ultimately discontinue methotrexate due to side effects [7, 8]. Use of biologic DMARDs is associated with an increased risk of infection as well as a significantly increased cost of treatment when compared to methotrexate [9, 10].

Of the children who require systemic treatment, many will reach a state of remission and some are able to discontinue their medications [11]. Unfortunately, there is a relatively high rate of flare after discontinuing systemic medications for children with both oligo and polyarticular disease [12]. While treatment options at the time of flare are essentially the same as at the time of disease onset (IAS vs systemic medications) there are no recommendations or data to support one treatment over another.

Physicians are likely to make treatment recommendations based on experience with this scenario or an attempt to accommodate patients’ preferences. Previous studies have shown that, of patients who were previously treated with systemic therapy then subsequently experience a disease flare after being in drug-free remission, most restart systemic medications while very few are treated with IAS [13, 14]. It is not clear that this immediate return to systemic treatment is the ideal approach to treatment especially in the context of patients who flare with only a few inflamed joints. A common reason to treat with IAS only, in addition to the rapid resolution of symptoms, is avoidance of systemic treatment. However, no data exists demonstrating that IAS can prevent the need to restart systemic therapy.

The primary aim of this study was to describe a population of patients with JIA who received a joint injection as monotherapy for treatment of arthritis after discontinuing a systemic DMARD and report on the probability of restarting systemic therapy within 12 months.

## Methods

To address our study aims, we analyzed data from an international registry, the Childhood Arthritis and Rheumatology Research Alliance (CARRA) Registry. Starting in July 2015, the Childhood Arthritis and Rheumatology Research Alliance (CARRA) began enrolling patients that met the International League of Associations for Rheumatology (ILAR) criteria for JIA and has grown to involve more than 70 member sites in the United States and Canada [15]. Eligible Registry patients must have been diagnosed with JIA prior to age 16 and be younger than 21 at the time of enrollment. Data are collected and uploaded to the Registry at the time of enrollment and then at study visits in the context of routine clinical care at 6 month intervals (+/− 3 months). Unscheduled study visits allow for data collection in the event of medication initiation. Clinic information is collected at each visit including patient/parent reported outcomes. Collection of active joint count (JC) did not include the specific joint(s) with active arthritis. Informed consent was obtained from all Registry participants.

Study patients included those with a primary rheumatologic diagnosis of JIA who were treated with and then discontinued methotrexate and/or a biologic DMARD. Patients who subsequently received an intraarticular joint injection without starting a DMARD (IAS group) or restarted either methotrexate or a biologic DMARD for treatment of JIA (systemic treatment group) were identified. Patients were excluded if the reason for restarting a systemic medication was uveitis or if they received both IAS and restarted a DMARD at the same visit. Patients with systemic JIA were also excluded due to the potential for medications to be restarted for extraarticular features of their disease. To assess clinical data at the time of arthritis flare, only patients whose joint injections or initiation of a DMARD coincided with study visit were included.

The success of IAS was inferred from the absence of subsequent DMARD use. This was assessed at the next two study visits following the IAS (i.e., approximately 6 and 12 months later). The primary outcome was the proportion of patients failing treatment with IAS and is reported along with 95% confidence intervals (CIs).

Statistical analyses were conducted using SAS 9.4 [Copyright (c) 2016 by SAS Institute Inc., Cary, NC, USA]. The study was approved by the Institutional Review Board at Penn State College of Medicine (STUDY00014208).

## Results

**Fig. 1:**
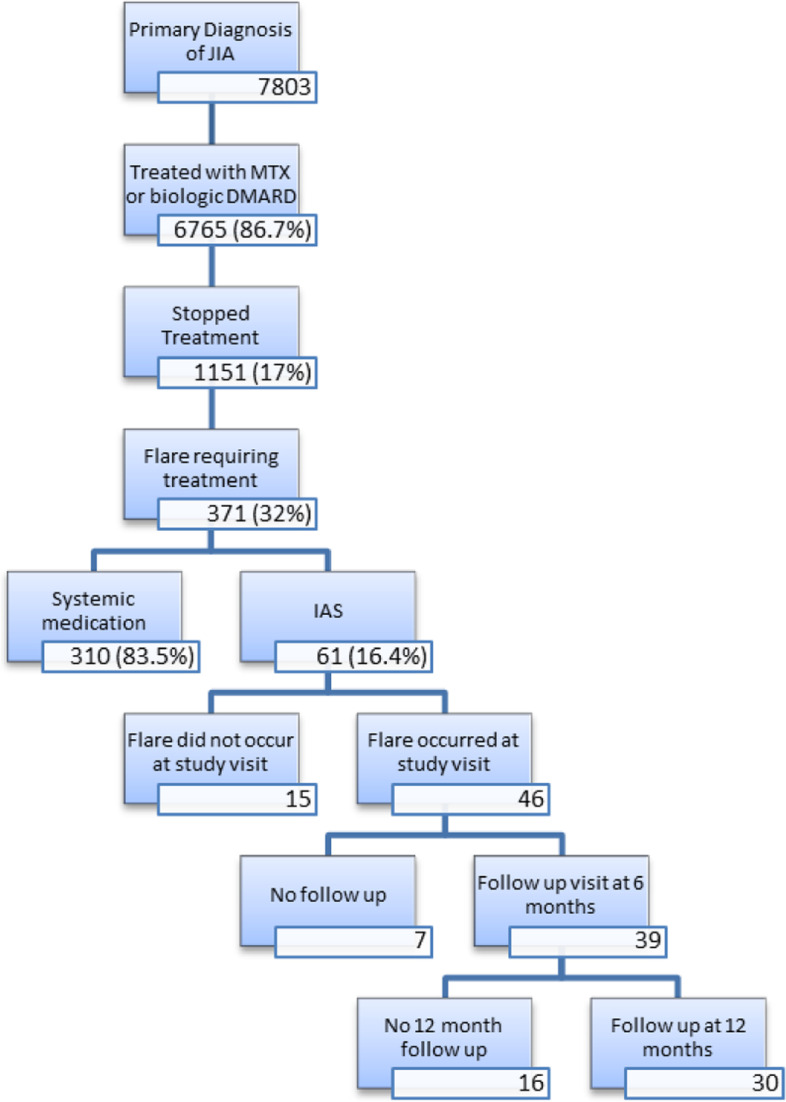
Flowchart showing numbers of subjects who met inclusion criteria and were treated with IAS at a study visit and then had follow up at 6 and 12 months

Data collected from the inception of the Registry (July 2015) to December 2019 were included. During that time the CARRA Registry had enrolled 7803 patients with JIA. There were 6765 patients (87%) who were treated with a systemic DMARD. Of those patients, 1151 (17%) discontinued systemic treatment during the course of their follow-up. There were 371 patients who either received an intraarticular steroid injection (61 patients) or restarted systemic treatment for treatment of JIA (310 patients). After excluding patients whose IAS injection or initiation of systemic therapy did not occur at a study visit, the total number of patients was 185 with 46 in the IAS group and 139 in the systemic treatment group. Characteristics of this group are presented in Table 1. At least 1 follow up visit occurred for 39 of the 46 patients in the IAS group and 2 follow up visits occurred for 30 of the subjects (Fig. 1).

**Table 1:** Demographic and clinic data for all patients requiring treatment with intraarticular steroids (IAS) or systemic treatment

Category		Total n (% or mean)	IAS n (%)	Systemic Treatment n (%)	***p***-value
	All	185 (100%)	46 (25%)	139 (75%)	
Sex	Male	46 (25%)	12 (26%)	34 (24%)	0.82
	Female	139 (75%)	34 (74%)	105 (76%)	
Age at Diagnosis (years)		7.3 (4.7)	7.1 (4.7)	7.4 (4.7)	0.72
Race*	White	160 (86%)	42 (91%)	118 (85%)	0.78
	Black, African American, African, or Afro-Caribbean	5 (3%)	2 (4%)	3 (2%)	
	Hispanic, Latino, or Spanish origin	14 (8%)	3 (7%)	11 (8%)	
	Asian	6 (3%)	0 (0%)	6 (4%)	
	Middle Eastern/North African	1 (1%)	1 (2%)	0 (0%)	
	Native American, American Indian or Alaskan Native	1 (1%)	0 (0%)	1 (1%)	
	Native Hawaiian or Other Pacific Islander	1 (1%)	0 (0%)	1 (1%)	
JIA subtype	Oligoarticular	77 (42%)	27 (59%)	50 (36%)	0.07**
	Polyarticular	78 (42%)	14 (30%)	64 (46%)	
	Psoriatic	15 (8%)	4 (9%)	11 (8%)	
	ERA	12 (6%)	1 (2%)	11 (8%)	
	Undifferentiated	3 (2%)	0 (0%)	3 (2%)	
ANA	Negative	98 (53%)	21 (46%)	77 (55%)	0.25
	Positive	87 (47%)	25 (54%)	62 (45%)	
RF	Negative	178 (96%)	46 (100%)	132 (95%)	0.2
	Positive	7 (4%)	0 (0%)	7 (5%)	
CCP	Negative	178 (96%)	45 (98%)	133 (96%)	0.68
	Positive	7 (4%)	1 (2%)	6 (4%)	
B27	Negative	171 (92%)	42 (91%)	129 (93%)	0.75
	Positive	14 (8%)	4 (9%)	10 (7%)	
Uveitis	No	159 (86%)	37 (80%)	122 (88%)	0.21
	Yes	26 (14%)	9 (20%)	17 (12%)	
Methotrexate	No	8 (4%)	3 (7%)	5 (4%)	0.41
	Yes	177 (96%)	43 (93%)	134 (96%)	
Biologic	No	49 (26%)	22 (48%)	27 (19%)	< 0.001
	Yes	136 (74%)	24 (52%)	112 (81%)	

*Patients were allowed to identify as more than 1 race thus the sum of percents is greater than 100. **p=0.0157 when psoriatic, enthesitis-related arthritis and undifferentiated arthritis were excluded

There were no differences in sex; age at diagnosis; history of uveitis; history of methotrexate use; or ANA, RF, CCP, or HLA-B27 status noted between the IAS and systemic treatment groups. The systemic treatment group had a higher proportion of patients who had previously been treated with at least one biologic DMARD (*p* < 0.001). The difference in the proportion of JIA subtypes was not different when all patients were included. However, if subtypes with small numbers (psoriatic, enthesitis-related, undifferentiated) were excluded and only oligoarticular and polyarticular JIA patients were included then the difference was significant (*p* < 0.05), with the systemic treatment group having a higher proportion of patients with polyarticular JIA. There was also no difference in the age at the time of flare between the two groups (13.1 for IAS vs 13.5 for systemic treatment, *p* = 0.57).

Clinical data showed that the systemic treatment group had a higher joint count, physician global assessment, cJADAS (clinical Juvenile Arthritis Disease Activity Score), and Childhood Health Assessment Questionnaire (CHAQ) score at the time of treatment. When the entire cohort of patients (*n* = 185) was restricted to patients with a joint count of one at the time of flare (*n* = 70), 50% of the patients (35/70) received systemic treatment despite there being no statistically significant difference in cJADAS at the time of flare (*p* = 0.3) compared to those that received IAS.

Of the 46 patients in the IAS group, 39 (85%) had 1 follow-up visit and 30 (65%) had 2 follow-up visits. The 7 patients with no 6 month follow-up had a lower physician global assessment than those with follow-up (1.5 vs 2.7, *p* < 0.05) while the 16 without a 12 month follow-up visit had lower patient global assessment (1.2 vs 2.8, *p* < 0.05) and a lower proportion with biologic exposure (25% vs 67%, p < 0.05). The remaining demographic and clinical variables for patients without follow up were not statistically significantly different.

The mean time to first and second follow-up visits was 5.4 months and 11.2 months, respectively. At the first follow-up visit, 19 patients restarted a systemic medication for treatment of JIA; thus the proportion restarting treatment at 6 months was 49% (95% CI: 32–65%). At the second follow-up visit 21 of the 30 patients had restarted systemic treatment giving a proportion restarting treatment by 12 months of 70% (95% CI: 51–85%) as shown in Table 2.

**Table 2:** Demographic and clinic data for the subset of patients treated with IAS (*n* = 39) and had at least once follow up comparing those who failed treatment with IAS and restarted a systemic medication with those who did not fail. RF, CCP, and B27 were left off due to the small number of positives. All were non-significant. *p > 0.05 when psoriatic, ERA and undifferentiated JIA were excluded

***6 month follow up***					
		**Total (n, %)**	**Failed n, %**	**No fail n, %**	**p-value**
	All	39, 100%	19, 49%	20, 51%	
Sex	Male	9, 23%	5, 26%	4, 20%	0.72
	Female	30, 77%	14, 74%	16, 80%	
Age at Diagnosis (years)		7.28 (4.59)	6.7 (3.8)	7.8 (5.2)	0.48
JIA subtype	Oligoarticular	22, 56%	10, 53%	12, 60%	0.63*
	Polyarticular	13, 33%	7, 37%	6, 30%	
	Psoriatic	3, 8%	1, 5%	2, 10%	
	ERA	1, 3%	1, 5%	0, 0%	
	Undifferentiated	0, 0%	0, 0%	0, 0%	
ANA	Negative	19, 49%	10, 53%	9, 45%	0.63
	Positive	20, 51%	9, 47%	11, 55%	
Uveitis	No	32, 82%	14, 74%	18, 90%	0.24
	Yes	7, 18%	5, 26%	2, 10%	
Methotrexate	No	1, 3%	0, 0%	1, 5%	1
	Yes	38, 97%	19, 100%	19, 95%	
Biologic	No	17, 44%	4, 21%	13, 65%	< 0.01
	Yes	22, 56%	15, 79%	7, 35%	
***12 month follow up***					
		**Total (n, %)**	**Failed n, %**	**No fail n, %**	**p-value**
	All	30, 100%	21, 70%	9, 30%	
Sex	Male	5, 17%	4, 19%	1, 11%	1
	Female	25, 83%	17, 81%	8, 89%	
Age at Diagnosis (years)		7.1 (4.5)	7.7 (4.2)	5.6 (5.1)	0.29
JIA subtype	Oligoarticular	16, 53%	11, 52%	5, 56%	1*
	Polyarticular	11, 37%	7, 33%	4, 44%	
	Psoriatic	2, 7%	2, 10%	0, 0%	
	ERA	1, 3%	1, 5%	0, 0%	
	Undifferentiated	0, 0%	0, 0%	0, 0%	
ANA	Negative	18, 60%	12, 57%	6, 67%	0.7
	Positive	12, 40%	9, 43%	3, 33%	
Uveitis	No	25, 83%	17, 81%	8, 89%	1
	Yes	5, 17%	4, 19%	1, 11%	
Methotrexate	No	1, 3%	0, 0%	1, 11%	0.3
	Yes	29, 97%	21, 100%	8, 89%	
Biologic	No	10, 33%	4, 19%	6, 67%	< 0.05
	Yes	20, 67%	17, 81%	3, 33%	

Among patients in the IAS group, those who failed by 6 months had a higher proportion of any prior exposure to a biologic DMARD (79% vs 35%, *p* < 0.01). A similar difference in the proportion of patients who had exposure to a biologic was also seen at 12 months (81% vs 33%, p < 0.05). Alternatively stated, for patients with no prior biologic use, the proportion that restarted systemic treatment after IAS was 24% (4/17) at 6 months and 40% (4/10) at 12 months, which is much lower than for patients with prior biologic use (68% at 6 months [p < 0.01] and 85% at 12 months [p < 0.05]) (Table 3).

**Table 3:** Proportion of patients who restarted systemic treatment after IAS injection

	6 months*	12 months**
Prior biologic use	0.68 (15/22)	0.85 (17/20)
No biologic	0.24 (4/17)	0.40 (4/10)
Total	0.49 (19/39)	0.7 (21/30)

*p < 0.01, ** p < 0.05

There were no statistically significant differences between those who failed IAS and those who did not fail with regards to sex, age at diagnosis, ANA/RF/CCP/B27 status, history of uveitis, or time since discontinuing last treatment. There were no statistically significant differences in the clinical data available at the time of IAS injection found between those who did and did not fail IAS for treatment of flare. There was a non-significant difference in joint counts at time of flare, with higher joint counts in those that failed treatment with IAS (*p* = 0.06 at 6 months, *p* = 0.09 at 12 months).

## Discussion

For children with JIA who flare after discontinuing treatment with a systemic medication, IAS injections are an option that may eliminate or delay the need to restart systemic medications. This study is the first to report on the proportion of patients who restart a systemic medication after being treated with IAS.

In our study, the majority of patients who required treatment after discontinuing a systemic medication were treated with a systemic medication (139/185) which is consistent with what has been reported by other groups [13, 14]. The higher cJADAS, joint count, and physician global assessment in this group treated with systemic medications suggest that disease activity at the time of a flare is a determinant of the treatment of choice. However, the finding that 50% of the patients with only a single joint involved were treated with systemic medication and that the cJADAS was not statistically significantly different between the two groups suggests that factors other than disease activity also contribute to the decision to use a systemic treatment. Physicians may have made treatment decisions based on the specific joint involved, availability of certain steroid preparations, and the success of prior injections in a given joint. Prior studies have demonstrated that these and other factors may contribute to the success of treatment with IAS [16–18]. It is also possible that the patients’ and/or caregivers’ familiarity with a previously used medication may ameliorate some of the hesitancy that is commonly encountered at the initiation of treatment with methotrexate or biologics.

Of the variables analyzed, only a history of biologic use was associated with failure of IAS. Biologics are typically used in patients who have either failed methotrexate or have clinical features at presentation that have been shown to predict methotrexate failure [1, 19, 20]. We suspect that the association of biologic use with IAS failure is indicative of the presence of a more aggressive disease process in a subset of patients which limits the clinical response to methotrexate and IAS.

Additionally, the observation that the distribution of JIA subtypes was not different between the group that failed IAS treatment for flare and those who did not fail seems to support the evolving hypothesis that the biologic phenotype may be more important for treatment decisions than the clinical phenotype in some patients [21]. The data do not allow us to determine whether the reason for IAS failure was due to arthritis resistant to IAS or accumulation of new joints after IAS. However, from a patient’s perspective this differentiation may not matter, since either scenario would lead to restarting systemic therapy.

The results of this analysis should be immediately impactful for pediatric rheumatologists as well as patients and their families. Patients who previously required a biologic DMARD for treatment of JIA who flare after discontinuing treatment should restart their biologic DMARD due to the high rate of failure of treatment with an IAS injection. Conversely, patients that were treated with methotrexate without the need for a biologic DMARD are good candidates for treatment with an IAS injection. This treatment approach would likely be well received by children and their families, especially considering the high frequency of methotrexate associated morbidity.

The greatest strength of this study is the large sample size contained within the CARRA Registry. There are some important limitations in this study. The Registry does not collect detailed information about medical decision making. There is also no indication of which joint is involved or which steroid dose and preparation were used for injections. Prior studies have shown variability in joint specific response to IAS with knees and wrists responding better than ankles and midfoot joints [18, 22]. Unfortunately, due to the lack of consistent availability of triamcinolone hexacetonide in the past decade, it is likely that some patients in this cohort received injections with the shorter-acting triamcinolone acetonide, potentially introducing bias into the results. Future studies should control for the specific joint, steroid preparation, and dose of steroid [23]. We also did not account for NSAID use given the frequent use of NSAIDs as needed, for brief periods of time, and for non-rheumatologic reasons (menstrual cramps, headaches etc.). Additionally, the CARRA Registry does not systematically collect data about NSAID use. The generalizability of these results are limited by the fact that the patients treated with IAS appeared to have less active disease at the time of flare. A limited number of patients had lab data available at the time of IAS injection which limited our ability to evaluate the differences in biomarkers, such as ESR. Nevertheless, this is the first report on this topic and these results should help facilitate discussion between patients, families and providers when faced with certain treatment decisions. While these results are informative, more data are needed to determine the most appropriate treatment of disease flares in patients with JIA flares who have been in a state of inactive disease.

In summary, a subset of patients with JIA who flare after discontinuing systemic medication may be successfully treated with IAS without needing to restart systemic medication. Patients with more benign disease course, indicated by the lack of a need for a biologic, may be better candidates for IAS treatment. Improved understanding of the biologic processes and heterogeneity in JIA may improve our ability provided targeted treatment.

List of abbreviations: JIA, Juvenile Idiopathic Arthritis. DMARD, Disease modifying anti-rheumatic drugs. IAS, Intraarticular steroid. CARRA, Childhood Arthritis Rheumatology Research Alliance. ILAR, International League Against Rheumatism. JC, Joint count. cJADAS, Clinical Juvenile Arthritis, Disease Activity Score. ANA, Antinuclear Antibodies, RF, Rheumatoid Factor. CCP, Cyclic Citrullinated Peptide.

## Data Availability

The data that support the findings of this study are available from CARRA but restrictions apply to the availability of these data, which were used under a data use agreement for the current study, and so are not publicly available. Data are however available from CARRA upon reasonable request (carragroup.org).
