# Correction: Intraarticular steroids as DMARD-sparing agents for juvenile idiopathic arthritis flares: Analysis of the Childhood Arthritis and Rheumatology Research Alliance Registry

**DOI:** 10.1186/s12969-022-00775-7

**Published:** 2022-12-14

**Authors:** Timothy Hahn, Carrie Daymont, Timothy Beukelman, Brandt Groh, Kimberly Hays, Catherine April Bingham, Lisabeth Scalzi, and for the CARRA Registry investigators

**Affiliations:** 1 Department of Pediatrics, Penn State Children’s Hospital, 500 University Dr, Hershey, 90 Hope Drive, P.O. Box 855, Hershey, PA 17033-0855 USA; 2 Department of Pediatrics, University of Alabama at Birmingham, CPPN G10, 1600 7th Ave South, Birmingham, AL 35233 USA

**Correction: Pediatr Rheumatol 20, 107 (2022)**

**https://doi.org/10.1186/s12969-022-00770-y**

The original publication of this article [1] contained an incorrect title. The updated title is available in this correction article, the original article has been updated.

## References

[1] Hahn T (2022). Intraarticular steroids as DMARD-sparing agents for juvenile idiopathic arthritis flares: Analysis of the Childhood Arthritis and Rheumatology Research Alliance Registry. Pediatr Rheumatol.

